# Expression of Antioxidant Defense Genes Determines Synergistic Ferroptosis Induction by the Combination of Erastin and Omega-3 Docosahexaenoic Acid in Prostate Cancer Cells

**DOI:** 10.1134/S1607672925601660

**Published:** 2026-02-04

**Authors:** M. O. Silkina, T. A. Kulagin, K. V. Klycheva, A. D. Shatsillo, M. D. Mastykina, A. V. Razumovskaya, K. M. Nyushko, S. V. Nikulin

**Affiliations:** 1 National Research University Higher School of Economics (HSE University), Moscow, Russia; 2 Lopatkin Research Institute of Urology and Interventional Radiology—Branch of the National Medical Research Radiological Center, Ministry of Health of the Russian Federation, Moscow, Russia; 3 Medical Institute of Continuing Education, Russian Biotechnological University, Moscow, Russia

**Keywords:** ferroptosis, erastin, prostate cancer, polyunsaturated fatty acids, docosahexaenoic acid

## Abstract

Ferroptosis is considered a promising strategy for inducing the death of tumor cells. However, the effectiveness of known ferroptosis inducers, such as erastin, is in some cases limited, which stimulates the search for new combined application strategies. In this study, the combined effect of erastin and docosahexaenoic acid (DHA) on prostate cancer cells was examined over time. It was shown that the combination of these agents is more toxic compared to their separate use for all tumor cells considered. At the same time, known ferroptosis inhibitors, ferrostatin-1 and deferoxamine, effectively prevented cell death, indicating the specificity of the mechanism of action. Transcriptomic analysis of cell lines differing in sensitivity to the combination revealed activation of antioxidant systems in more resistant cells (in particular, pronounced expression of the *NQO1* and *GCLM* genes responsible for the reduction of quinones to hydroquinones and the synthesis of glutathione, respectively). The obtained results indicate the high synergistic potential of the erastin–DHA combination for ferroptosis induction and open new possibilities for the development of combined approaches to the therapy of resistant tumors.

## INTRODUCTION

Ferroptosis is a form of programmed cell death discovered in 2012 that differs from apoptosis, necrosis, and other known types of cell death [1]. The key features of ferroptosis include iron dependence and lipid peroxidation, which leads to damage to cell membranes and subsequent death [1]. In recent years, ferroptosis has attracted increasing attention of oncology researchers, because it was shown that many drug-resistant tumor cells are especially sensitive to this type of cell death [2]. This opens new prospects for the development of alternative strategies for treating malignant neoplasms, especially in cases of resistance to traditional treatments, which is a particularly pressing problem in the context of prostate cancer [3].

Ample data on the sensitivity of various tumor cell lines to ferroptosis inducers, particularly to the canonical ferroptosis inducer erastin, has accumulated in the scientific literature [4, 5]. However, the majority of studies have focused on the effects of individual compounds, whereas the potential for the combined use of various agents to enhance ferroptosis is insufficiently studied. Too few studies have been devoted to investigating the synergistic effect between the conventional ferroptosis inducers and biologically active compounds. However, there is already evidence indicating the effectiveness of this approach. For example, it was recently shown that vitamin C increases the sensitivity of pancreatic cancer cells to erastin-induced ferroptosis [6].

A promising candidate for studying the combined induction of ferroptosis is docosahexaenoic acid (DHA), an omega-3 polyunsaturated fatty acid. Previously, DHA was shown to induce cell death in various tumor lines via the ferroptotic mechanism [7, 8]. Interestingly, DHA induces ferroptosis in tumor cell lines via a mechanism different from that of erastin [9]. This makes DHA a promising candidate for studying its combined effect with erastin, since their different mechanisms of action could potentially lead to a pronounced synergistic effect in inducing ferroptosis. Interestingly, several studies have examined the antitumor effect of the combined treatment with DHA and erastin; however, the results obtained in these studies are inconsistent with each other. In one case, the use of DHA resulted in an increased toxic effect on tumor cells when used in combination with erastin [10], whereas in another, there was no statistically significant effect from the use of this combination [11]. All of above makes a more detailed study of the combined effects of erastin and docosahexaenoic acid a relevant issue.

In this study, we examined the combined effect of the classical ferroptosis inducer erastin and docosahexaenoic acid on prostate cancer cells. In addition to determining the viability of cell lines after treatment with different agents and their combinations, we also examined the kinetics of cell death for the first time. The aim of the study was to determine whether the combined use of these agents leads to a consistently more pronounced toxic effect compared to their separate use in various cell lines, as well as to investigate the molecular mechanisms that underlie the sensitivity and resistance of cells to this drug combination. The obtained results are relevant for the development of new approaches to the treatment of resistant tumors.

## MATERIALS AND METHODS

Prostate cancer cell lines (PC-3 and DU145) were cultured in DMEM/F12 medium (PanEco, Russia) supplemented with 10% FBS (Capricorn, Germany), 1% Glutamax (Gibco, United States), and 1% Anti-anti (Gibco, United States) at 37°C and 5% CO_2_ in a cell incubator (Sanyo, Japan). Cell cultures were passaged using 0.25% trypsin-EDTA solution (PanEco, Russia) every 2–3 days. Cell growth dynamics were assessed visually using a ZOE Fluorescent Cell Imager inverted microscope (Bio-Rad, United States).

To analyze the kinetics of ferroptosis, cells were plated in a 96-well plate at a density of 10 000 cells per well and incubated in 100 μl of culture medium in a cell incubator (37°C, 5% CO_2_) for 24 h. Then, the culture medium was replaced with the control medium or the medium containing 5 μM erastin and 25 μM DHA (Sigma-Aldrich, United States) for the DU145 line and 200 μM for the PC-3 line, 100 μM deferoxamine (DFO, Acros Organics, United States) or 0.5 μM ferrostatin-1 (Sigma-Aldrich, United States), as well as their combinations. The concentrations of erastin and DHA were selected based on the previously obtained data on the sensitivity of various lines (data not shown). The cells were incubated at 37°C and 5% CO_2_ for 24 h. To visualize cell death, the added medium also contained propidium iodide (PI, Lumiprobe, Russia) at a concentration of 1 μg/mL. The red fluorescent signal could only be detected after cell membrane disruption. Therefore, the cell death kinetics were determined by counting the number of red fluorescent objects (dead cells) over time using the IncuCyte® S3 Live-Cell Analysis System (Sartorius, United States). The imaging was performed in the red channel fluorescence mode with simultaneous phase imaging (PI). Four non-overlapping fields of view were recorded in each well, together covering approximately 50% of the well area of a 96-well plate. The number of PI-positive (dead) cells was counted automatically using the built-in Red Object Count algorithm in IncuCyte® software. For each time interval, the mean value was calculated across the four fields of view for each well. The obtained values were used to plot cell death dynamics curves.

Cell viability was analyzed using the MTT assay after 24 h of incubation with the preparations during the analysis of cell death kinetics. For this purpose, the medium in the wells of the plate was replaced with a medium containing 10% MTT solution (PanEco, Russia) in DPBS (PanEco, Russia) at a concentration of 5 mg/mL. The plates were incubated for 2 h, after which the cells were lysed with a solution containing 10% sodium dodecyl sulfate, 50% dimethylformamide, and acetic acid (pH 4.7) [12].

Experiments on cell treatment with the drugs and their combinations were performed in three technical replicates. Experiments on assessment of cell death kinetics and viability were performed in three biological replicates. The statistical significance of differences was estimated using analysis of variance (ANOVA).

Transcriptomic data for the cell lines were obtained from the GSE108545 dataset [13]. Data processing and visualization of results were performed in R environment version 4.5.1. Differential gene expression was assessed using the DESeq2 software package [14]. Signaling pathway enrichment was determined by gene set enrichment analysis (GSEA) using the fgsea package and the Hallmark and Canonical Pathways collections from the MSigDB database [15–18]. Correction for multiple comparisons was performed using the Benjamini–Hochberg procedure; changes with *p*adj < 0.05 were considered statistically significant. Differential expression was visualized using a volcano plot (EnhancedVolcano package), and the most enriched pathways were presented as scatter plots (dotplot) based on the ggplot2 package [19, 20].

## RESULTS AND DISCUSSION

In this study, we successfully compared the viability of two prostate cancer cell lines (DU145 and PC-3) treated with erastin, DHA, and their combination. Furthermore, the possibility of preventing cell death as a result of exposure to known ferroptosis inhibitors, such as ferrostatin-1 (an antioxidant that scavenges free radicals) and DFO (an iron ion chelator), was investigated, and the cell death kinetics under the considered conditions were determined for the first time.

**Fig. 1.**
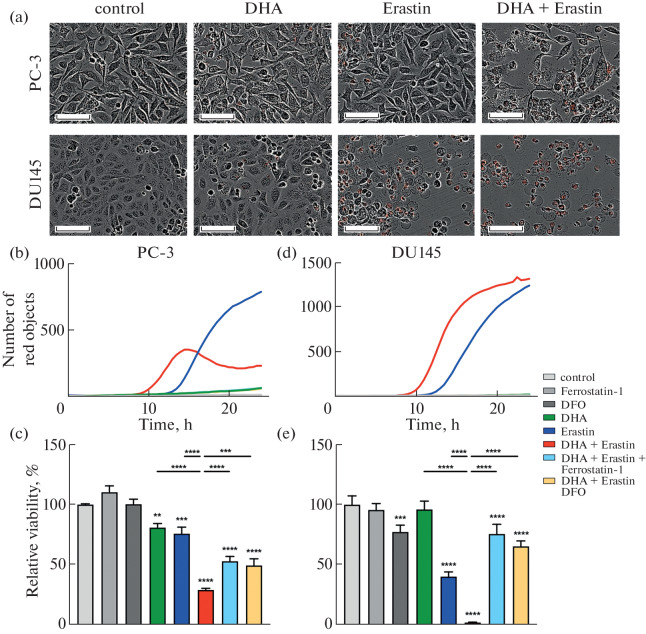
(a) Merged phase-contrast and fluorescence images of PC-3 and DU145 cells after 24 h of incubation under different conditions: control, DHA (25 μM for DU145 and 200 μM for PC-3), erastin (5 μM), and their combination (DHA + erastin). Red objects indicate the cells that have lost membrane integrity. Scale bar 100 μm. Kinetics of cell death (number of red objects) over time under the indicated conditions for PC-3 (b) and DU145 (d) cells, and (c, e) relative cell viability after 24 h of treatment with erastin, DHA, ferrostatin-1 (0.5 μM), and DFO (100 μM), applied alone or in combination. ** *p* < 0.01, *** *p* < 0.001, **** *p* < 0.0001.

The obtained data demonstrate a pronounced synergistic effect of the combined use of erastin and DHA on prostate cancer cells (Fig. 1a). According to IncuCyte data (Figs. 1b, 1d), cell death began significantly earlier in the case of the combined use of erastin and DHA compared to erastin and DHA alone. For example, it is clearly seen that, in both cell lines, the difference in the time of beginning of active cell death when treated with the combination of drugs versus erastin alone is approximately 3–4 h. The treatment with DHA alone did not cause cell death during the time period analyzed. The results of the MTT assay (Figs. 1c, 1e) are consistent with the results obtained using IncuCyte: cell viability was significantly reduced when the combination of erastin and DHA was used, particularly in the case of the DU145 cell line, for which the combination reduced viability to nearly zero.

Important confirmation of the specificity of the induced cell death is the fact that the addition of ferrostatin-1 and deferoxamine, known inhibitors of ferroptosis, protected cells from the action of the combination of erastin and DHA, according to both IncuCyte (Figs. 1b, 1d) and MTT (Figs. 1c, 1e) assays. This confirms the key role of ferroptosis in the effect and demonstrates the versatility of this mechanism in different prostate cancer cells.

It should be noted that partial discrepancies between the results of IncuCyte and MTT assay may be due to differences in the operating principles of these methods. For example, in addition to cell death itself, the results of the MTT assay may be affected by processes such as slowed cell growth or decreased metabolic activity, which do not immediately lead to cell death. As a result, the MTT assay may be less specific for detecting cell death induction than recording death dynamics using IncuCyte. However, in general, both methods showed consistency in assessing the effectiveness of the combination, which increases the significance of the data obtained.

It should be noted that, in other publications, the synergistic effect of the combination of erastin and DHA was not always detected [11], which is likely due to differences in experimental conditions, primarily in the models and concentrations used. Regarding the last parameter, it is important to note that significantly lower concentrations of DHA (10–20 μM) were used in the aforementioned study, whereas in the present study, concentrations of 25 and 200 μM were used for DU145 and PC-3 cells, respectively. Despite the apparently high concentration of DHA in the last case, it is worth noting that the level of 200 μM can be reached in human blood plasma, although it approaches the upper limit of possible values [21].

**Fig. 2.**
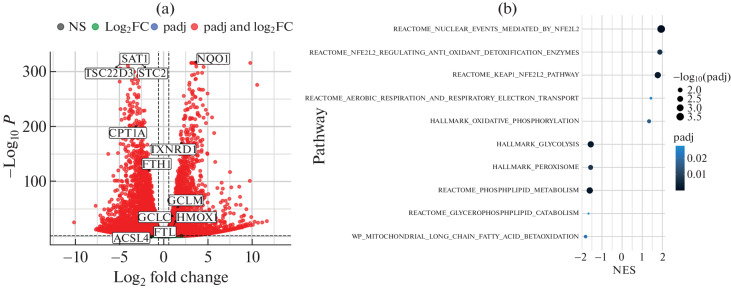
Transcriptomic differences between cell lines with varying sensitivity to combined treatment with erastin and docosahexaenoic acid (more resistant PC-3 compared to more sensitive DU145). (a) Volcano plot showing differential gene expression. (b) Gene set enrichment analysis (GSEA) of the most significant pathways. NES, normalized enrichment score.

To elucidate the molecular mechanisms underlying the observed differences in the efficiency of the combination of erastin and DHA, transcriptome profiles of the cell lines were analyzed using the GSE108545 dataset [13] (Fig. 2). The results showed that the relative resistance of PC-3 cells compared to DU145 cells may be due to the initial features of the transcriptome profile that create a state of increased readiness for oxidative stress. For example, PC-3 cells showed an increased expression of the *NQO1*, *HMOX1*, and *TXNRD1* genes, as well as the *GCLC* and *GCLM* genes. The majority of these genes were characterized as important components of the response to ferroptotic oxidative stress [9] (Fig. 2a). The *NQO1* gene, whose expression changed most significantly, encodes the enzyme NAD(P)H-quinone oxidoreductase 1, which is involved in the reduction of quinones to hydroquinones. The *GCLC* and *GCLM* genes encode subunits of the enzyme glutamate-cysteine ligase, which catalyzes the first and rate-limiting stage in the synthesis of glutathione, a key cellular antioxidant. Moreover, it is worth noting that all of these genes are targets of the Nrf2 transcription factor (*NFE2L2* gene). Activation of the Nrf2-regulated antioxidant defense transcriptional program in PC-3 cells compared to DU145 cells was additionally confirmed using gene set enrichment analysis (Fig. 2b).

Another important observation was the decreased expression of the *ACSL4* and *CPT1A* genes in PC-3 cells (Fig. 2a). The ACSL4 protein is responsible for the activation of fatty acids by converting them into acyl-CoA, whereas the CPT1A protein is required for the transport of fatty acids into mitochondria for their beta-oxidation. The pathway enrichment analysis also revealed a general suppression of lipid metabolism pathways, including the biosynthesis and remodeling of membrane phospholipids, with a simultaneous increase in the activity of the respiratory chain and mitochondrial oxidative phosphorylation (Fig. 2b). This switch in cellular metabolism from predominant utilization of lipids to more active utilization of glucose limits the incorporation of polyunsaturated fatty acids into phospholipids and reduces the formation of lipid peroxides, key substrates of ferroptosis. As a result of this metabolic shift, PC-3 cells become significantly more resistant to the effects of DHA and the combination of DHA and erastin. Thus, the initial changes in the PC-3 transcriptome, including a metabolic shift towards increased mitochondrial respiration, limitation of lipid peroxide accumulation, and activation of antioxidant systems, create a state that is less susceptible to erastin-induced ferroptosis in the presence of high docosahexaenoic acid concentrations.

Thus, this is the first study to demonstrate the kinetic features of the synergistic effect of erastin and DHA in prostate cancer cells. The obtained results confirm that the combination of erastin and DHA can significantly enhance the induction of ferroptosis in prostate cancer cells, and this effect is realized even when individual agents have a low toxicity. Effective protection by ferroptosis inhibitors additionally confirms the specificity of the mechanism of action. For the first time, the possible molecular mechanisms underlying the observed differences in the effectiveness of the combination of erastin and DHA have been proposed based on transcriptomic data. The analysis showed that the relative resistance of the PC-3 cell line compared to DU145 is apparently associated with the activation of antioxidant systems. In particular, the more resistant line showed a pronounced expression of the *NQO1* gene, which encodes an enzyme involved in the reduction of quinones to hydroquinones, as well as the *GCLM* and *GCLC* genes, which are responsible for the synthesis of glutathione, a key cellular antioxidant. However, further experiments are required to confirm the direct contribution of these genes to the observed resistance. In addition, a metabolic shift toward decreased lipid beta-oxidation activity was detected, with a simultaneous suppression of the processes that ensure the synthesis of substrates from which lipid peroxides are formed. However, even for the relatively resistant PC-3 prostate cancer cell line, the addition of DHA significantly increases the efficacy of the conventional ferroptosis inducer erastin. Thus, the considered approach may be a promising strategy for overcoming drug resistance in prostate cancer and deserves further study.
